# Alcohol accelerates the development of esophageal squamous cell carcinoma through elevated Gram-negative bacteria in peripheral circulation

**DOI:** 10.1186/s40164-025-00617-8

**Published:** 2025-02-25

**Authors:** Zehua Zhang, Le Kang, Yanfen Gu, Zhuyun Leng, Tao Chen, Meidong Xu

**Affiliations:** 1 Endoscopy Center, Department of Gastroenterology, Shanghai East Hospital, Tongji University School of Medicine, No. 150 Jimo Road, Pudong New District, Shanghai, 200120 China; 2 Department of Endoscopy, Fudan University Shanghai Cancer Center, Shanghai, China

**Keywords:** Blood microbiome, Gram-negative bacteria, Alcohol, ESCC

## Abstract

Alcohol consumption is intricately linked to the incidence of esophageal squamous cell carcinoma (ESCC). This study comprehensively investigates the role of alcohol-induced microbial alterations in ESCC progression. A retrospective analysis of 328 patients demonstrated that alcohol consumption markedly increases the risk of ESCC and boosts the expression of the proliferation marker Ki67. Patients with alcohol-related ESCC exhibited substantially higher blood microbiome diversity, characterized by the dominance of Gram-negative bacteria, and elevated serum lipopolysaccharides (LPS) levels. In a mouse model, alcohol consumption not only augmented tumor burden but also compromised gut barrier integrity, facilitating bacterial translocation. Significant elevations in Gram-negative bacteria, such as *Bacteroidales* in the blood and *Escherichia coli* in esophageal tissues, were observed. Mechanistically, alcohol and LPS synergistically activated pro-inflammatory pathways, including TNF, TLR, NF-κB, and MAPK, which fueled ESCC cell proliferation. Meanwhile, LPS triggered necroptosis in normal esophageal epithelial cells. These findings reveal that alcohol-induced microbial dysbiosis in peripheral circulation and LPS-mediated inflammatory responses form a novel pathogenic mechanism in ESCC. Targeting Gram-negative bacteria and LPS could provide a promising therapeutic strategy for managing alcohol-related ESCC. Further research is urgently warranted to explore the interaction between microbial changes and the tumor microenvironment.

**Supplementary Information:**

The online version contains supplementary material available at 10.1186/s40164-025-00617-8.

**To the Editor**,

Alcohol consumption is strongly associated with the incidence of esophageal squamous cell carcinoma (ESCC) [1, 2]. Recent research has highlighted the close relationship between the esophageal microbiota and esophageal cancer [3]. A comparative analysis of the microbiota in esophageal paracancerous tissue from alcohol-related and non-alcohol-related ESCC patients revealed a significant decrease in microbial abundance in alcohol-consuming patients, with a notable increase in Gram-negative bacteria [4]. Additionally, several studies have shown that alcohol can increase colonic epithelial barrier permeability, allowing bacterial products to enter the bloodstream [5, 6].

Our study retrospectively analyzed data from 328 patients with esophageal mucosal lesions. Spearman correlation analysis indicated that alcohol consumption, types of alcohol (especially spirit), volume of alcohol, and duration of drinking were positively correlated with the occurrence of ESCC, tumor count, invasion depth, and Ki67 expression in tumors (Fig. 1A). Binary logistic regression analysis, with ESCC and intraepithelial neoplasia as dependent variables, showed that the regression coefficient for alcohol consumption was 1.851 (z = 2.654, *p* = 0.008, OR 6.367, 95%CI 1.623–24.984), indicating that alcohol consumption increases the risk of ESCC and intraepithelial neoplasia by 6.367 times (Fig. 1B). Stepwise regression analysis further demonstrated a positive correlation between alcohol consumption and ESCC Ki67 expression (Fig. 1C). The blood microbiome of patients with non-alcoholic ESCC and alcoholic ESCC was examined using 2bRAD-M, a novel technique for detecting microbiome in human body fluids [7]. The results showed significantly increased blood microbiome diversity, abundance of Gram-negative bacteria, and levels of Gram-negative *Alphaproteobacteria* in the alcoholic ESCC group (Fig. 1D-G). Lipopolysaccharides (LPS), the primary type of bacterial endotoxins, were also elevated in the blood of alcoholic ESCC patients (Fig. 1J). Next, we used a 4-Nitroquinoline 1-oxide (4-NQO)-induced ESCC mouse model and administered 10% alcohol in drinking water at different stages (Supplementary Fig. 1A). The results showed that mice in the non-early 8-week alcohol-drinking group had more tumors and elevated serum LPS levels (Fig. 1K-O). Staining results showed larger esophageal tumors and increased Ki67-positive cells in non-early 8-week alcohol-drinking groups (Fig. 2A, B and Supplementary Fig. 1B-D). Additionally, alcohol intake downregulated the tight junction proteins zonula occludens-1 (ZO-1) and Occludin in mouse colon, although this effect was not as pronounced in the esophagus (Supplementary Fig. 1E).

**Fig. 1:**
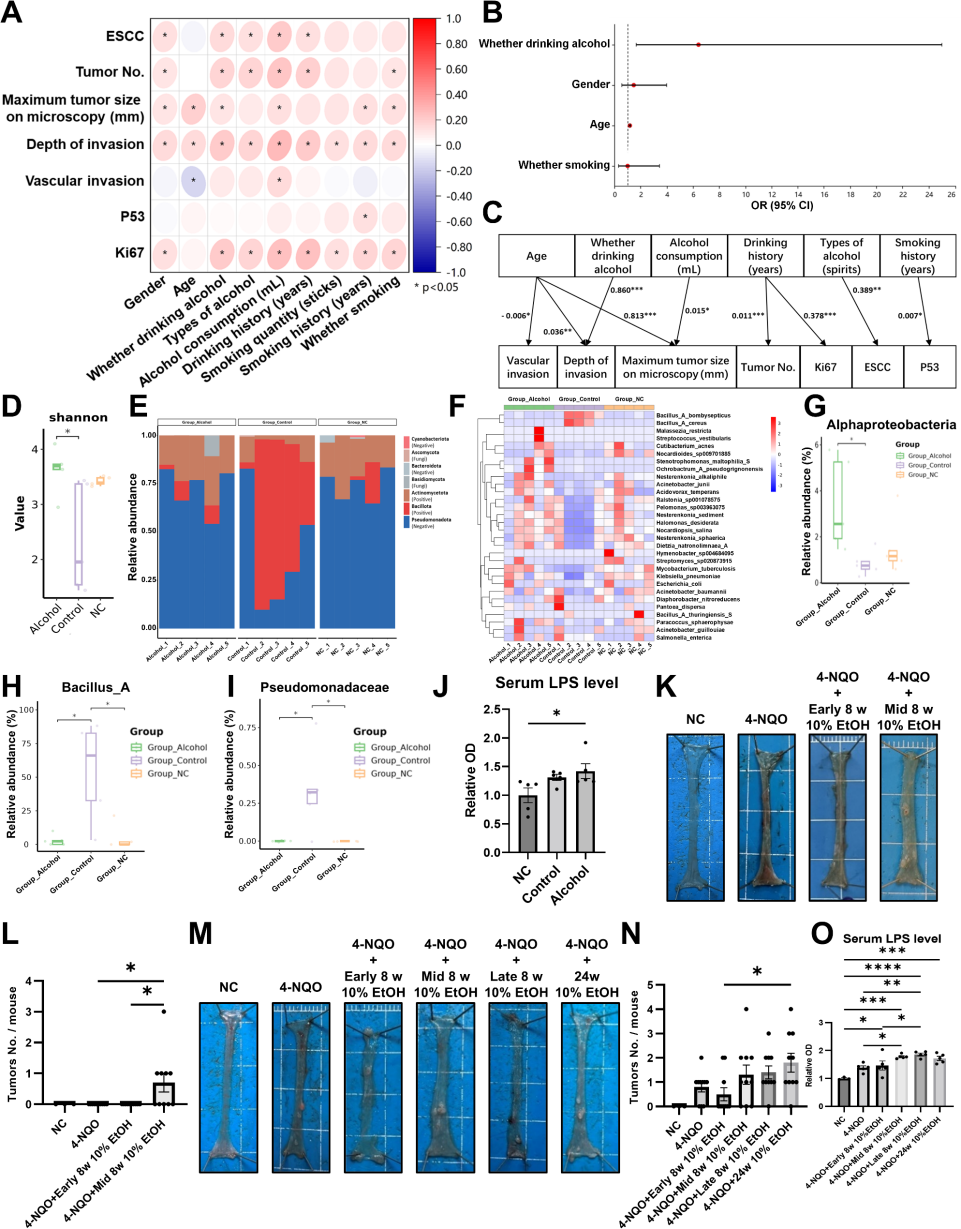
Alcohol consumption promotes the growth of ESCC through Gram-negative bacteria and LPS. Data from 328 patients with esophageal mucosal lesions were analyzed. (**A**) Spearman correlation analysis, (**B**) binary logistic regression analysis (X-axis represent odds ratio (OR) and 95% confidence intervals (95% CI), and Y-axis represent risk factors), and (**C**) stepwise regression analysis was used to calculate the pathological conditions and patient conditions. The types of alcohol include beer, Chinese yellow wine and red wine, spirits, spirits with others. 2bRAD-M simplified metagenomic sequencing was performed on the blood from non-alcoholic ESCC patients (Control group), alcoholic ESCC patients (Alcohol group), and patients with colon polyps which served as negative control group (NC group) (*n* = 5). (**D**) microbial alpha diversity, (**E**) microbial distribution at the phylum level (with Gram-positive and Gram-negative labeled), (**F**) heat map of microbial enrichment at the species level, and (**G**-**I**) the microbial comparison difference maps were analyzed. (**J**) The patients’ serum LPS levels of three groups were compared. A 4-Nitroquinoline 1-oxide (4-NQO) induced mouse ESCC model was established and 10% alcohol (EtOH) drinking water was given to the mice at different times. (**K**, **M**) Mice were sacrificed at the end of 16 and 24 weeks and photos of esophagus were shown. (**L**, **N**) The number of tumors in the mice in 16-week cycle (*n* = 10, NC *n* = 3) and 24-week cycle (*n* = 10, NC *n* = 3) was compared. (**O**) The serum LPS level of the mice was detected in 24-week cycle (*n* = 5, NC *n* = 3). X-axis represent grouping and Y-axis represent shannon value, relative abundance, relative optical density, or tumors number. *P < 0.05; **P < 0.01; ***P < 0.001; ****P < 0.0001

**Fig. 2:**
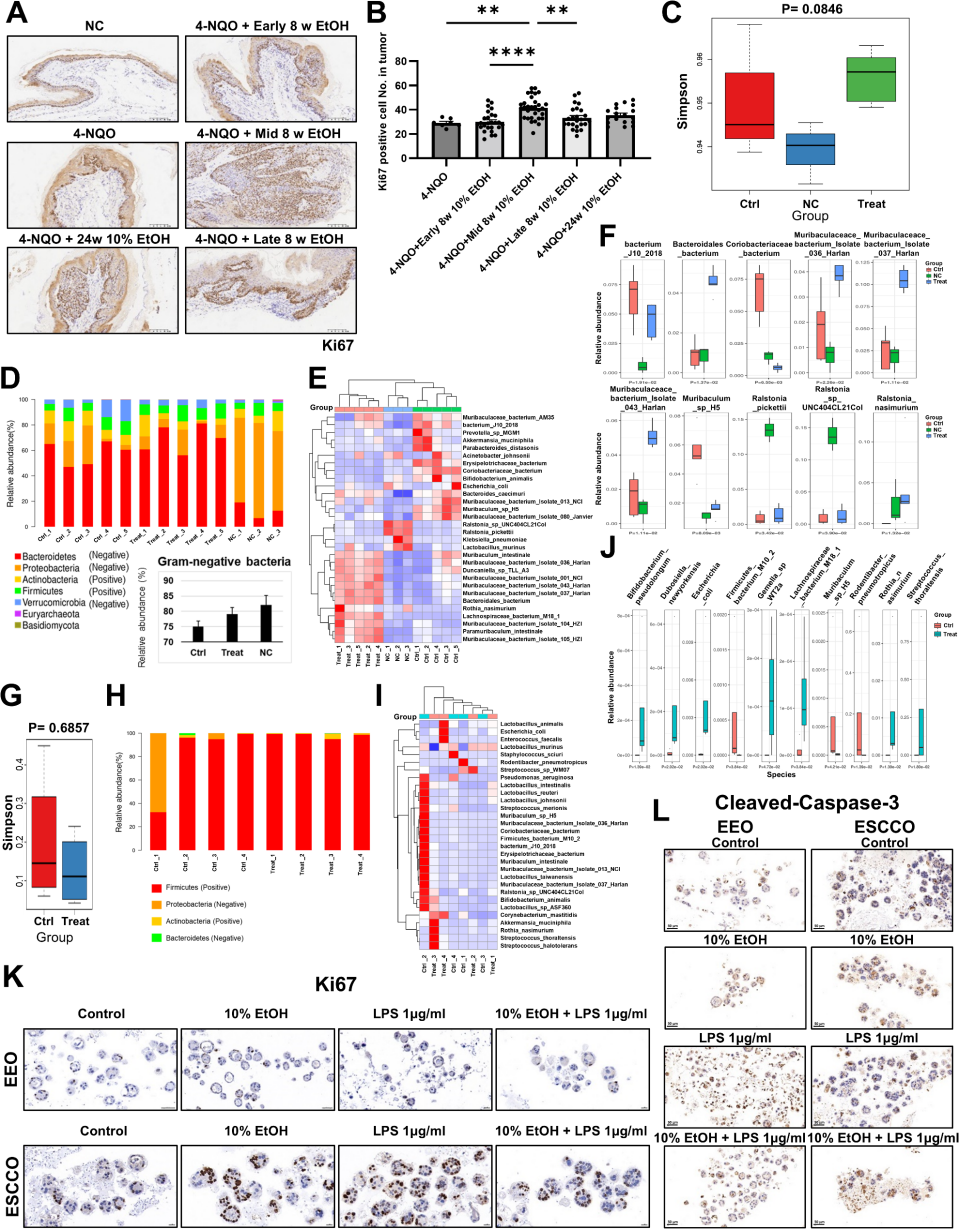
Alcohol consumption affects ESCC proliferation, the colonic barrier, blood microbiota, and esophageal microbiota in mice. (**A**) Ki67 staining of the esophagus of mice sacrificed at 24 weeks, and (**B**) the count of Ki67-positive cells in the tumor was compared, 4-NQO *n* = 7, early 8 weeks *n* = 26, middle 8 weeks *n* = 31, late 8 weeks *n* = 24, 24 weeks *n* = 18. Blood from normal mice (NC), mice drinking alcohol at middle 8 weeks of 16 weeks cycle (Treat), and non-drinking model mice (Ctrl) (*n* = 5, NC *n* = 3) were sequenced by 2bRAD-M simplified metagenomic sequencing, and (**C**) microbial alpha diversity, (**D**) microbial distribution at the phylum level and the proportion of Gram-negative bacteria, and (**E**) species-level heat map were analyzed. (**F**) A comparative difference map of the three groups of microorganisms was drawn. The esophageal microbial conditions of the mice from above groups (*n* = 4) were detected, and (**G**) the microbial alpha diversity, (**H**) phylum-level community abundance, (**I**) species-level heat map, and (**J**) microbial comparative difference map was analyzed. Esophageal epithelial organoids (EEO) and ESCC organoids (ESCCO) were stimulated with 10% alcohol and/or 1 µg/ml LPS for 48 h, and (**K**) Ki67 staining and (**L**) cleaved-caspase-3 staining were performed. X-axis represent grouping and Y-axis represent Ki67 positive cell number, simpson value, or relative abundance. *P < 0.05; **P < 0.01; ***P < 0.001; ****P < 0.0001

Further investigation using 2bRAD-M detection in the blood and esophagus of model mice (16-week cycle) revealed that alcohol consumption increased blood microbiome diversity and the abundance of Gram-negative bacteria (Fig. 2C-E), as well as elevated levels of Gram-negative *Bacteroidales bacterium* and *Muribaculaceae* (Fig. 2F). In contrast, alcohol decreased esophageal microbiome diversity in alcohol-drinking mice (Fig. 2G), while the level of Gram-negative *Escherichia coli* increased (Fig. 2H-J). RNA-sequencing of mouse esophagus showed upregulation of tumor necrosis factor (TNF) and mammalian target of rapamycin (mTOR) pathways in the alcohol-drinking group (Supplementary Fig. 1F), with increased Ki67, cell death, and several related pathways (Supplementary Fig. 1G). Mouse esophageal epithelial organoids (EEO) and ESCC organoids (ESCCO) were used to demonstrate that alcohol or LPS from *Escherichia coli* O111:B4 enhanced the proliferation of ESCCO, while LPS promoted the death of EEO (Fig. 2K, L and Supplementary Fig. 1H). RNA-sequencing showed increased TNF pathway and Ki67 in ESCCO compared to EEO (Supplementary Fig. 2A, F). Alcohol upregulated the TNF pathway and Ki67 in ESCCO (Supplementary Fig. 2B, F), and LPS enhanced the TNF and nuclear factor-kappa B (NF-κB) pathways in both ESCCO and EEO (Supplementary Fig. 2C, E, F). Notably, LPS alone upregulated necroptosis in EEO (Supplementary Fig. 2E, F). Thus, alcohol and LPS promote ESCC proliferation, while LPS promotes the death of normal cells (Supplementary Fig. 2G).

The higher baseline expression of the TNF pathway in cancer cells may be the primary reason for the dual effects of alcohol and LPS. Both the TNF and toll-like receptor (TLR) pathways regulate the NF-κB and mitogen-activated protein kinase (MAPK) pathways [8]. The mTOR pathway is regulated by the TNF, TLR, and MAPK pathways [9, 10]. Although the interactions between these pathways remain unclear, the pivotal role of the TNF pathway in ESCC is evident. Consistent with our findings, a study reported that LPS can promote the death of normal oral squamous cells and the proliferation of oral squamous cell carcinoma, which can be explained by the mechanisms identified in our study [11]. Furthermore, a recent study found changes in the fecal microbial profiles of ESCC patients, with increased fecal Gram-negative bacteria and blood LPS [12]. Building on this foundation, our study used 2bRAD-M to detect the human blood microbiome and combined mouse models and cell experiments to investigate the underlying mechanisms, emphasizing the synergistic effects of alcohol and LPS in promoting ESCC development. In terms of pathogenesis, the low expression of aldehyde dehydrogenase in the esophageal epithelium means that accumulated acetaldehyde cannot be decomposed [13]. Acetaldehyde induces oxidative damage, DNA adduct formation, and metabolism changes, ultimately promoting ESCC [13]. Both oral and gut microorganisms can convert alcohol into acetaldehyde, which is another major pathogenic pathway besides LPS and warrants further research [14].

Our study has some limitations. In real life, people are exposed to alcohol much more frequently than to 4-NQO. To further clarify the impact of drinking on the occurrence and development of ESCC, future studies should conduct 4-NQO-induced ESCC experiments in mice that are either drinking or abstaining from alcohol. Additionally, the microbiomes of humans and mice are significantly different, and our study could not identify specific pathogenic microorganisms. The role of tumor microenvironment cells in promoting tumor growth under conditions of elevated Gram-negative bacteria and LPS also remains to be explored.

In conclusion, our study provides novel evidence that alcohol intake increases the abundance of Gram-negative bacteria in the peripheral circulation of ESCC patients. Based on the upregulation of the TNF pathway in tumor cells, alcohol and LPS synergistically promote ESCC cell proliferation, while LPS promotes the death of normal squamous cells, ultimately accelerating ESCC development. This study suggests that targeting Gram-negative bacteria and LPS, a novel mechanism distinct from acetaldehyde, may be a potential therapeutic target for alcohol-related ESCC.

## Electronic supplementary material

Below is the link to the electronic supplementary material.

Supplementary Material 1

Supplementary Material 2

Supplementary Material 3

Supplementary Material 4

## Data Availability

The authors confirm that data supporting the findings of this study are available within the article or upon request from the corresponding author. The raw sequence data reported in this paper have been deposited in the Genome Sequence Archive (Genomics, Proteomics & Bioinformatics 2021) in National Genomics Data Center (Nucleic Acids Res 2022), China National Center for Bioinformation / Beijing Institute of Genomics, Chinese Academy of Sciences (GSA: CRA021313，CRA021379 and CRA021375) that are publicly accessible at https://ngdc.cncb.ac.cn/gsa.

## Abbreviations

4-NQO: 4-Nitroquinoline 1-oxide
EEO: Esophageal epithelial organoids
ESCC: Esophageal squamous cell carcinoma
ESCCO: ESCC organoids
LPS: Lipopolysaccharides
MAPK: Mitogen-activated protein kinase
mTOR: Mammalian target of rapamycin
NF-κB: Nuclear factor-kappa B
TLR: Toll-like receptor
TNF: Tumor necrosis factor
ZO-1: Zonula occludens-1
