# Supplementary figures and images for: Alcohol accelerates the development of esophageal squamous cell carcinoma through elevated Gram-negative bacteria in peripheral circulation
Source: Exp Hematol Oncol. 2025 Feb 25;14:19. doi: 10.1186/s40164-025-00617-8 (PMC11863458; doi:10.1186/s40164-025-00617-8)

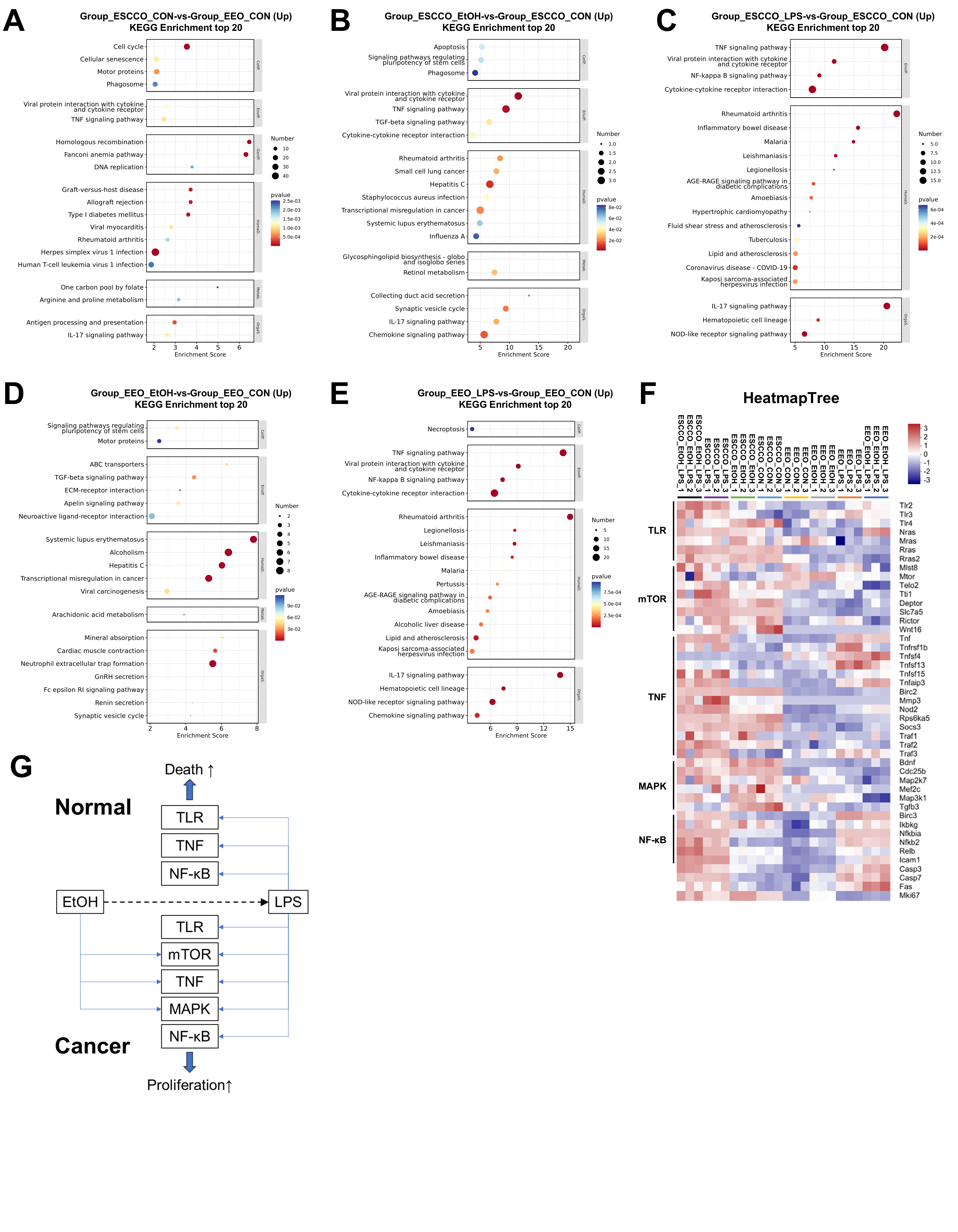

Supplement: Supplementary file 1 — Supplementary Material 1 [file 40164_2025_617_MOESM1_ESM.tif]

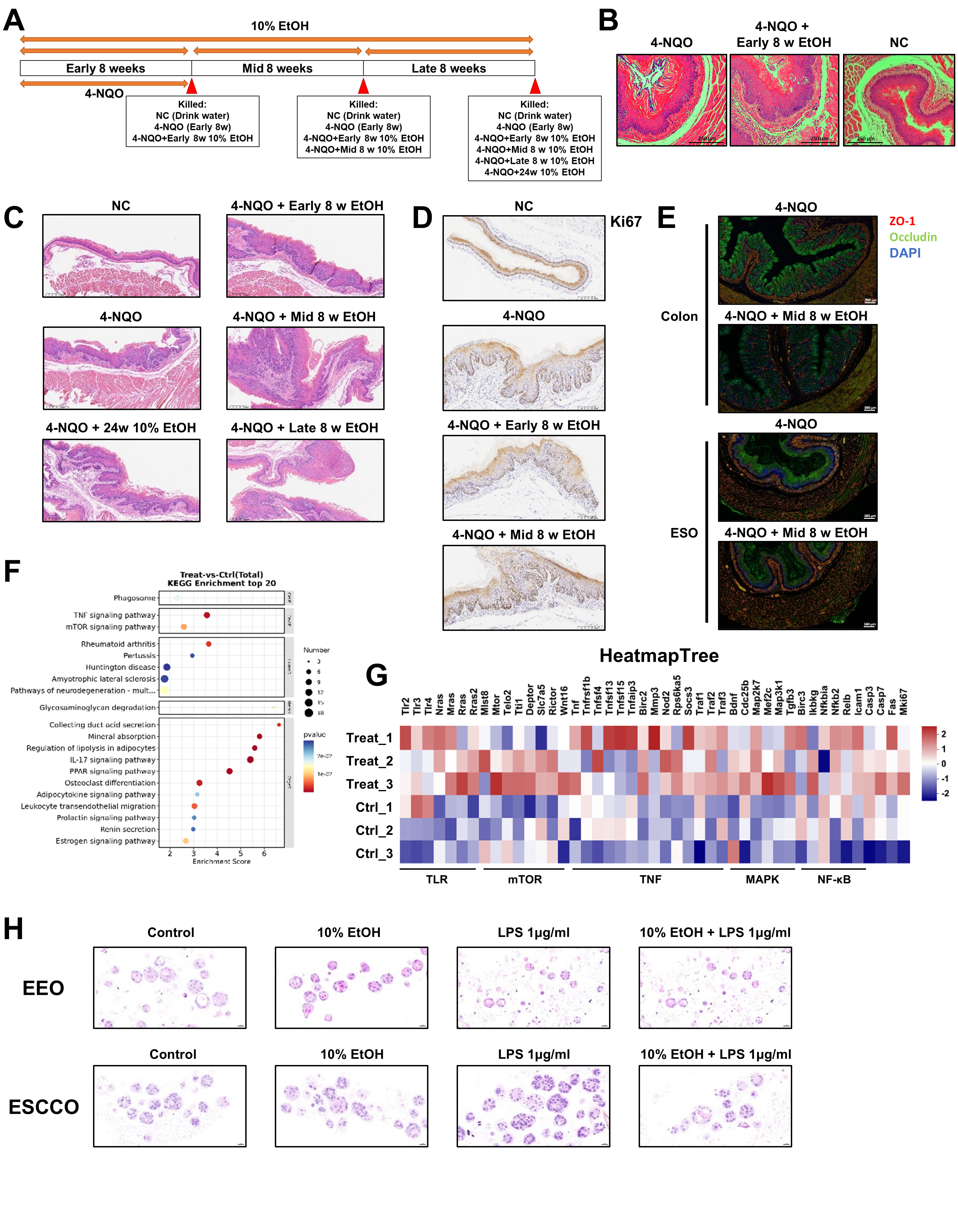

Supplement: Supplementary file 2 — Supplementary Material 2 [file 40164_2025_617_MOESM2_ESM.tif]
