# Supplementary material for: Alcohol accelerates the development of esophageal squamous cell carcinoma through elevated Gram-negative bacteria in peripheral circulation
Source: Exp Hematol Oncol. 2025 Feb 25;14:19. doi: 10.1186/s40164-025-00617-8 (PMC11863458; doi:10.1186/s40164-025-00617-8)
Supplement: Supplementary file 3 — Supplementary Material 3 [file 40164_2025_617_MOESM3_ESM.docx]

**Supplementary Figure Legends**

**Supplementary Figure 1.** Alcohol and LPS regulate the growth and death balance of esophageal squamous cells and ESCC. (A) Animal experiment flow chart. (B, C) H&E staining of the esophagus of mice sacrificed at 8 weeks and 24 weeks. (D) Ki67 staining of the esophagus of mice sacrificed at 16 weeks. (E) ZO-1 (red) and Occludin (green) staining of colon and esophagus of mice sacrificed at 16 weeks. RNA sequencing was performed on the esophagus of ESCC mice that drank alcohol for 8 weeks (4-NQO + Mid 8 w EtOH, 16 weeks, n=3) and ESCC mice that did not drink alcohol (4-NQO, 16 weeks, n=3), and (F) KEGG differential enrichment maps and (G) gene expression heat maps were generated. EEO and ESCCO were stimulated with 10% alcohol and/or 1μg/ml LPS for 48 hours, and (H) H&E staining was performed.

**Supplementary Figure 2.** Alcohol and LPS regulate the TNF, TLR, MAPK, NF-κB, and mTOR pathways of esophageal squamous cells and ESCC. RNA sequencing was performed on the EEO and ESCCO stimulated with 10% alcohol and/or 1μg/ml LPS for 48 hours, and (A-E) KEGG differential enrichment maps and (F) gene expression heat maps were generated. (G) Diagram of the mechanism by which alcohol and LPS promote ESCC.
