# Supplementary material for: Alcohol accelerates the development of esophageal squamous cell carcinoma through elevated Gram-negative bacteria in peripheral circulation
Source: Exp Hematol Oncol. 2025 Feb 25;14:19. doi: 10.1186/s40164-025-00617-8 (PMC11863458; doi:10.1186/s40164-025-00617-8)
Supplement: Supplementary file 4 — Supplementary Material 4 [file 40164_2025_617_MOESM4_ESM.docx]

**Supplementary Materials and Methods**

Patient data analysis

We retrospectively analyzed data from 328 patients with esophageal mucosal lesions since 2019 from Endoscopy Center (Department of Gastroenterology, Shanghai East Hospital, Tongji University School of Medicine, Shanghai, China). Using correlation analysis, we investigated the relationships between ESCC, depth of invasion (M1-3 and SM1-3), maximum tumor size on microscopy (mm), Ki67, and P53 with nine factors: age, gender, alcohol consumption, type of alcohol (beer, Chinese yellow wine and red wine, spirits, spirits with others), alcohol consumption amount (mL), drinking history (years), smoking status, smoking quantity (sticks), and smoking history (years). The strength of these relationships was expressed using Spearman's correlation coefficient. Further, we performed a binary logistic regression analysis using age, gender, alcohol consumption status, and smoking status as independent variables, and esophageal tumor (ESCC and intraepithelial neoplasia) as the dependent variable. A total of 319 samples were included, with 9 samples excluded due to missing data. First, we evaluated the overall effectiveness of the model that the model quality remains the same regardless of the inclusion of independent variables. A p-value of less than 0.05 indicated that the included independent variables were valid. The formula for the model is: ln(p/1-p) = -6.561 + 1.851 * alcohol consumption status + 0.352 * gender + 0.125 * age - 0.040 * smoking status (where p represents the probability of esophageal tumor presence, and 1-p represents the probability of its absence). Finally, we performed stepwise regression analysis, using age, gender, alcohol consumption, type of alcohol (beer, Chinese yellow wine and red wines, spirits, spirits with others), alcohol consumption amount (mL), drinking history (years), smoking status, smoking quantity (sticks), and smoking history (years) as independent variables, and the pathological results ESCC, depth of invasion (M1-3 and SM1-3), maximum tumor size on microscopy (mm), Ki67, and P53 as dependent variables respectively. After automatic model selection, only the significant variables remained in the model, and the R-squared value was calculated. The model passed the F-test, indicating its validity. The formula for the model is: Dependent variable = constant regression coefficient + independent variable regression coefficient * independent variable. We also tested for multicollinearity in the model, finding that all VIF values were below 5, indicating no multicollinearity issues. Additionally, the D-W value was close to 2, suggesting no autocorrelation, meaning there was no relationship between the sample data. Therefore, the model was robust. The analysis was conducted using SPSS 27.0. The collection of patients data and blood was approved by the Ethics Committee of East Hospital Affiliated to Tongji University (Permit Number: 2024YS-149). Blood from patients with colon polyps without other diseases served as a negative control group (NC) to exclude the interference of other factors on the results, n=5. Blood from Alcoholic ESCC patients and non-alcoholic ESCC patients served as Alcohol group and Control group, n=5.

DNA extraction and 2bRAD-M analysis

DNA was extracted from mouse esophageal tissue or mouse blood and human blood samples used the DNeasy Blood & Tissue Kit (QIAGEN, Shanghai, China). The 2bRAD-M library preparation was based on the original protocol developed by Wang et al [1]. DNA was digested using 4 U of the BcgI enzyme (BioLabs, New England). Adapters were ligated to DNA fragments. DNA was extracted after amplification, PCR, and excision of a band of approximately 100 bp by QIAquick PCR Purification Kit (QIAGEN, Shanghai, China). Sample-specific barcodes were introduced through PCR using platform-specific barcode-bearing primers. PCR products were purified and sequenced on the Illumina Nova PE150 platform. The 2bRAD-M process was conducted by Qingdao OE Biotech Co., Ltd. (Qingdao, China). A total of 173,165 microbial genomes were downloaded from the NCBI RefSeq database. Restriction fragments were sampled from these genomes, creating a comprehensive 2bRAD microbial genome database. The set of 2bRAD tags sampled from each genome was assigned a GCF number. All 2bRAD tags were then compared with those from other genomes. Species-specific 2bRAD markers were developed from these tags, collectively forming a 2bRAD marker database. To control for false positives in species identification, a G score was calculated for each species. The G score is the harmonic mean of the read coverage of 2bRAD markers for a species and the total number of 2bRAD markers possible for that species. A G score threshold of 5 was used to limit false-positive discoveries [2]. The average read coverage of all 2bRAD markers for each species was then calculated, representing the number of individuals of that species in the sample at the given sequencing depth. Finally, the relative abundance of a species was determined by calculating the ratio of the number of individuals of that species to the total number of individuals from all detectable known species in the sample.

4-NQO-induced mouse ESCC model

Six-week-old female C57BL/6J mice (Model Organisms Center, Shanghai, China) were used for modelling and administered 100 µg/mL 4-NQO (N8141, Sigma-Aldrich, St. Louis, MO) in their drinking water for 8 weeks, with changed weekly. After 8 weeks, 4-NQO was removed, and the mice were given regular drinking water for an additional 0 or 8 or 16 weeks, making the total period 8 or 16 or 24 weeks. The modeling process was divided into 3 stages: the early 8 weeks (weeks 1-8, with 10% ethanol in drinking water), the middle 8 weeks (weeks 9-16, with 10% ethanol), the late 8 weeks (weeks 17-24, with 10% ethanol), and the full 24 weeks (weeks 1-24, with 10% ethanol throughout). The groups were: 8 weeks cycle: NC (Drink water), 4-NQO (Early 8w), 4-NQO+Early 8w 10% EtOH; 16 weeks cycle: NC (Drink water), 4-NQO (Early 8w), 4-NQO+Early 8w 10% EtOH, 4-NQO+Mid 8 w 10% EtOH; 24 weeks cycle: NC (Drink water), 4-NQO (Early 8w), 4-NQO+Early 8w 10% EtOH, 4-NQO+Mid 8 w 10% EtOH, 4-NQO+Late 8 w 10% EtOH, 4-NQO+24w 10% EtOH. Body weight was measured weekly. Mice were sacrificed at weeks 8, 16 and 24, with blood collected from the heart apex which used for LPS measurement or 2bRAD-M. The esophagus was dissected for examination and staining [3, 4]. The animal research was performed under the protocol that has been approved by the Biological Research Ethics Committee of the Tongji University (TJBB05523101).

Esophageal epithelial organoid and ESCC organoid culture

The entire esophagus of the mouse was isolated, with the mucosal layer or ESCC tissue separated and cut into 1-2 mm fragments. After wash with PBS (Biosharp, Beijing, China), tissues were incubated by Trypsin-EDTA (0.05%)(Thermo Fisher, New York, USA) for 1 hour and dramatically washed 3-5 times with a wash buffer (advanced DMEM/F12 (Life Technologies, Scotland, UK) containing 10% fetal bovine serum (FBS) (Thermo Fisher) and 1% penicillin/streptomycin (Thermo Fisher)) to collect the single cells in supernatant. The cells were then resuspended in 600 µL ice-cold reduced growth factor Matrigel (Discovery Labware, Bedford, USA) with 600 μL EEO culture medium (A 1:1 mixture of advanced DMEM/F12 medium and WRN conditional medium included 1 × GlutaMAX (Thermo Fisher), 1 × B27 supplement (Thermo Fisher), 10 mM nicotinamide (Yeasen, Shanghai, China), 10 μM SB202190 (Yeasen, Shanghai, China), 50 ng/mL EGF (Yeasen, Shanghai, China), 500 nM A83-01 (Yeasen, Shanghai, China), 10μM Y-27632 (Yeasen, Shanghai, China) and 1% penicillin/streptomycin), and plated in the center of a 24-well plate, followed by the addition of 500 µL of EEO culture medium [5]. Mature organoids were used for 1μg/ml LPS (*Escherichia coli* O111:B4, L2630, Sigma-Aldrich, St. Louis, MO) or 10% alcohol (EtOH) stimulation experiments for 48 hours.

RNA-sequencing and analysis

RNA was extracted from mouse esophageal tissues and organoids after grinding, and the extracted RNA was used for RNA sequencing and analysis. Total RNA was extracted using the TRIzol reagent (Invitrogen, CA, USA) according to the manufacturer’s protocol. RNA purity and quantification were evaluated using the NanoDrop 2000 spectrophotometer (Thermo Scientific, USA). RNA integrity was assessed using the Agilent 2100 Bioanalyzer (Agilent Technologies, Santa Clara, CA, USA). Then the libraries were constructed using VAHTS Universal V6 RNA-seq Library Prep Kit according to the manufacturer’s instructions. The transcriptome sequencing and analysis were conducted by OE Biotech Co., Ltd. (Shanghai, China). For specific details, please refer to our previous study [6].

Immunostaining and Histology

Mouse esophageal tissues and organoids were fixed, dehydrated, and paraffin-embedded for preservation, followed by sectioning, deparaffinization, and hydration for various staining procedures. Specific methods are as described in our previous study [6, 7]. The details of antibody: Anti-ki67 (Abcam, ab16667); Cleaved Caspase-3 (Asp175) (Cell Signaling Technology, 9664); Anti- ZO-1 (Cell Signaling Technology, 13663); Anti- Occludin (Cell Signaling Technology, 68534).

LPS measurement

We used the ToxinSensor™ Chromogenic LAL Endotoxin Assay Kit (L00350, GenScript, Nanjing, China) to measure LPS levels in the serum of patients and mice. The procedure was performed according to the manufacturer's instructions, and the final results were presented as fold change.

Statistical analysis

All results are presented as the mean ± standard error of the mean (SEM). The number (n) quoted throughout the manuscript refers to the number of patients or mice. The t-test was used to compare two groups, and one-way ANOVA and Tukey’s multiple comparisons test were used to compare more than two groups to determine the statistical significance (GraphPad Prism V9): *P < 0.05, **P < 0.01, ***P < 0.001, and ****P < 0.0001. Please refer to Patient data analysis for clinical analysis.

**References**

1. Wang S, Meyer E, McKay JK, Matz MV. 2b-RAD: a simple and flexible method for genome-wide genotyping. Nat Methods. 2012;9(8):808-10.

2. Sun Z, Huang S, Zhu P, Tzehau L, Zhao H, Lv J, et al. Species-resolved sequencing of low-biomass or degraded microbiomes using 2bRAD-M. Genome biology. 2022;23(1):36.

3. Tang XH, Knudsen B, Bemis D, Tickoo S, Gudas LJ. Oral cavity and esophageal carcinogenesis modeled in carcinogen-treated mice. Clinical cancer research : an official journal of the American Association for Cancer Research. 2004;10(1 Pt 1):301-13.

4. Yao J, Cui Q, Fan W, Ma Y, Chen Y, Liu T, et al. Single-cell transcriptomic analysis in a mouse model deciphers cell transition states in the multistep development of esophageal cancer. Nature communications. 2020;11(1):3715.

5. Ko KP, Zhang J, Park JI. Establishing transgenic murine esophageal organoids. STAR Protoc. 2022;3(2):101317.

6. Zhang Z, Yan X, Kang L, Leng Z, Ji Y, Yang S, et al. TRPM8 inhibits substance P release from primary sensory neurons via PKA/GSK-3beta to protect colonic epithelium in colitis. Cell Death Dis. 2024;15(1):91.

7. Sun M, Li Z, Wang X, Zhao M, Chu Y, Zhang Z, et al. TAOK3 Facilitates Esophageal Squamous Cell Carcinoma Progression and Cisplatin Resistance Through Augmenting Autophagy Mediated by IRGM. Adv Sci (Weinh). 2023;10(29):e2300864.
